# Age Related Bioenergetics Profiles in Isolated Rat Cardiomyocytes Using Extracellular Flux Analyses

**DOI:** 10.1371/journal.pone.0149002

**Published:** 2016-02-12

**Authors:** Kennedy S. Mdaki, Tricia D. Larsen, Lucinda J. Weaver, Michelle L. Baack

**Affiliations:** 1 Children’s Health Research Center, Sanford Research, Sioux Falls, SD, United States of America; 2 Sanford School of Medicine-University of South Dakota, Sioux Falls, SD, United States of America; 3 Children’s Health Specialty Clinic, Sanford Children’s Hospital, Sioux Falls, SD, United States of America; Mount Sinai School of Medicine, UNITED STATES

## Abstract

Mitochondrial dysfunction is increasingly recognized and studied as a mediator of heart disease. Extracellular flux analysis (XF) has emerged as a powerful tool to investigate cellular bioenergetics in the context of cardiac health and disease, however its use and interpretation requires improved understanding of the normal metabolic differences in cardiomyocytes (CM) at various stages of maturation. This study standardized XF analyses methods (mitochondrial stress test, glycolytic stress test and palmitate oxidation test) and established age related differences in bioenergetics profiles of healthy CMs at newborn (NB1), weaning (3WK), adult (10WK) and aged (12–18MO) time points. Findings show that immature CMs demonstrate a more robust and sustained glycolytic capacity and a relative inability to oxidize fatty acids when compared to older CMs. The study also highlights the need to recognize the contribution of CO_2_ from the Krebs cycle as well as lactate from anaerobic glycolysis to the proton production rate before interpreting glycolytic capacity in CMs. Overall, this study demonstrates that caution should be taken to assure that translatable developmental time points are used to investigate mitochondrial dysfunction as a cause of cardiac disease. Specifically, XF analysis of newborn CMs should be reserved to study fetal/neonatal disease and older CMs (≥10 weeks) should be used to investigate adult disease pathogenesis. Knowledge gained will aid in improved investigation of developmentally programmed heart disease and stress the importance of discerning maturational differences in bioenergetics when developing mitochondrial targeted preventative and therapeutic strategies for cardiac disease.

## Introduction

Cardiovascular disease (CVD) is the leading cause of death in the United States claiming up to 1 in every 4 deaths [1]. Mitochondrial dysfunction is now recognized as one of the key mediators in the pathophysiology of cardiac disease [2] presenting increased opportunity to develop innovative diagnostic, preventative and therapeutic strategies for CVD [3–6]. The ability to investigate and develop these applications using accurate, translatable animal or cell models is key to ongoing progress in this field. Bioenergetics profiling through real-time extracellular flux analysis (XF) has gained recognition as a powerful tool to understanding metabolic mechanisms of disease [7]. However, using and interpreting XF analyses of cardiomyocytes (CM) in the context of cardiac health and disease requires improved understanding of normal cellular bioenergetics at various stages of maturation, particularly for the field of developmental programming. Establishing normal bioenergetics profiles of healthy CMs at different developmental stages warrants thorough investigation and is the primary objective of this study.

The heart requires a constant supply of ATP in order to maintain continuous contractile function. To meet this demand, the heart can utilize a wide variety of metabolic fuels depending on supply and demand [8]. Specifically, fuel preference switches at some point during normal development. The fetal heart relies predominantly on anaerobic glycolysis to meet ATP demand [9]. This is likely due to a relatively hypoxic *in utero* environment [10] which does not favor mitochondrial driven aerobic respiration. However, after birth the placenta is no longer available to provide a continuous supply of nutrients and oxygen supply rapidly increases with the shift from fetal to neonatal circulation. Mitochondrial oxidation, especially from fatty acids (FA), is a primary source of energy in the normal, resting adult heart [8, 9, 11]. Validated XF methods that account for maturational differences in cardiac glycolytic and respiratory capacity over time are not well established [9]. For various reasons, cardiac metabolism is often interrogated using XF technology on neonatal CMs [12–14] which may not reflect metabolic characteristics of the aging heart [9]. In order to better understand the role of cellular bioenergetics in the development of cardiac health and disease, this study used XF analyses to delineate normal metabolic profiles of CMs at various maturational stages and standardized an approach to compare metabolic differences over time. This knowledge is critical to advance research focused on mitochondrial dysfunction in the heart, particularly when it relates to developmentally programmed cardiovascular disease.

## Materials and Methods

### Animal Care

This study followed guidelines set forth by the Animal Welfare Act, The National Institutes of Health Guide for the Care and Use of Laboratory Animals and was approved by the Institutional Animal Care and Use Committee. Animals were housed in a temperature-controlled, light-dark cycled facility with free access to water and chow. Male and female Sprague-Dawley rats (Harlan Laboratories Inc., Indianapolis, IN) were fed a standard rat diet (Teklad Diet 2018, Harlan Laboratories, Madison, WI) throughout the study.

### Primary cardiomyocyte isolation

Primary neonatal CMs were isolated from 2–3 Sprague Dawley rat litter mates within the first 24 hours after birth (NB1). Animals were anesthetized with 5% isoflurane and cervical dislocation was performed. Hearts were removed and immediately transferred to a Hank’s Balanced Salt Solution (HBSS). The atria were removed and disposed. The ventricles were minced and digested in a mixture of 0.1% Trypsin and 0.02% DNase (in 0.15M NaCl) while stirred in sterile glass bottles for 5 min at 50rpm. Next, the cells were triturated gently (at approximately 1ml/1sec) for 5 minutes. Supernatant was transferred to a 50ml tube containing bovine serum through a cell strainer. The above was repeated until the hearts were completely digested. The mixture of bovine serum and cells was centrifuged at 1600rpm at 22°C for 10 minutes (Eppendorf Centrifuge 5810 R). Supernatant was removed and the pellet re-suspended in 1ml DMEM-1 (supplemented with 10% bovine serum albumin and 1% Penicillin/Streptomycin) together with 0.0002% DNAse. Cells were plated to an uncoated 35mm dish and incubated for 1 hour in 5% CO_2_ at 37°C. Next, CMs were gently detached from the dish, re-suspended in the DMEM-1, and counted leaving behind the rapidly attaching fibroblasts. CMs were plated at a seeding density of 150,000 cells/well in 0.1% gelatin coated seahorse V7-PS microplates using DMEM-2, which is essentially DMEM-1 supplemented with 100μM 5-bromo-2’-deoxyuridine to inhibit proliferation of non-myocytes as previously reported [15]. Cardiomyocytes were incubated overnight at 5% CO_2_ 37°C.

To isolate CMs from older rats (beyond the NB1 time point) the following method was used. Rats were anesthetized with isoflurane/O_2_ mix. The heart was harvested and immediately placed in iced perfusion buffer (PB) consisting of 120.4mM NaCl, 14.7mM KCl, 0.6mM KH_2_PO_4_, 0.6mM Na_2_HPO_4_, 1.2mM MgSO_4_-7H_2_O, 10mM Na-Hepes, 4.6mM NaHCO_3_, 30mM Taurine, and 5.5mM Glucose. While still beating, the heart was cannulated and perfused using the Langendoff apparatus with PB for 5 minutes followed by digestion with collagenase II (Worthington Biochem, Lakewood, NJ). Once digested, the heart was cut from the cannula just below the atria and the ventricles teased into 10–12 small pieces. Ventricular sections were further separated and cells were transferred to stopping buffer (PB supplemented with 12.5μM CaCl_2_ and 1% Bovine serum albumin) in a 15ml sterile tube. Cell number and quality were checked under microscope before proceeding and then allowed to sediment by gravity for 10 minutes at room temperature before calcium re-introduction. Cells were transferred to an uncoated 35mm dish and 0.25mM CaCl_2_ was added, swirled gently and left to sit for 4 min. Calcium re-introduction was done using 0.25mM, 0.5mM, 0.5mM and 0.3mM CaCl_2_ in order to produce calcium-tolerant CM for plating [16]. The cells were counted using a TC20 automatic cell counter with trypan blue detection to determine cell viability and number simultaneously. CMs were resuspended in culture media (MEM) and plated on laminin (18μg/ml) coated seahorse V7-PS cell culture microplates and allowed to attach for at least 1 hour.

### Extracellular Flux Analyses

**Fig 1:**
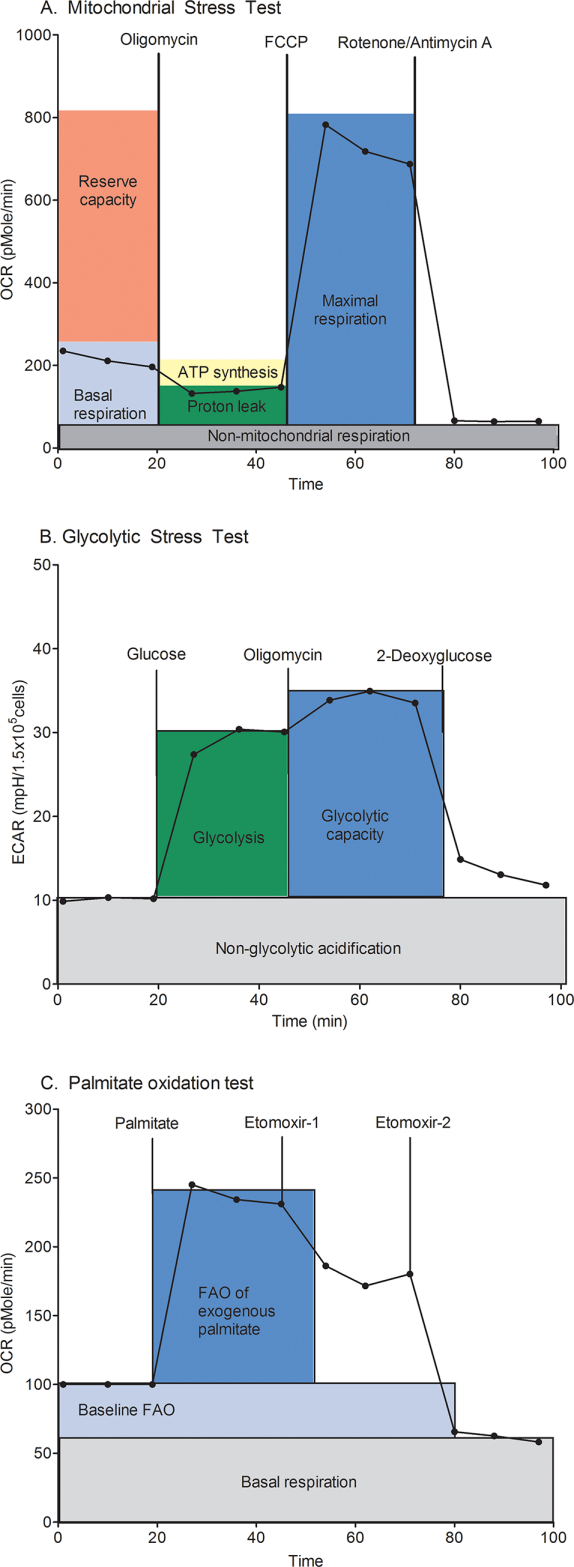
XF Analyses of Isolated Cardiomyocytes. Examples of real-time trace oxygen consumption rate (OCR) and extracellular acidification rate (ECAR) during extracellular flux analyses using (A) mitochondrial stress test, (B) glycolytic stress test and (C) palmitate oxidation test. Data points represent mean of three readings taken at each time-point. Drug injections are shown for each test and boxes visually depict computed data.

Mitochondrial stress test, glycolytic stress test and palmitate oxidation assay were completed on primary isolated CMs from each litter by real-time extracellular flux analyses using a Seahorse XF24 flux analyzer (Seahorse Bioscience, North Billerica, MA) as illustrated in Fig 1. Specifically, oxygen consumption rate (OCR) and extracellular acidification rate (ECAR) were measured under the following specified conditions for each maturational time-point.

#### Mitochondrial stress test

OCR, an indicator of mitochondrial respiration was measured using a Seahorse XF24 analyzer. Cells were washed with XF assay medium (Seahorse Biosciences, 100965–000) supplemented with 10mM Glucose and 1mM Pyruvate and placed in a 37°C incubator without CO_2_ for an hour. An automated Seahorse XF24 protocol consisted of 11 min calibration/equilibration, followed by synchronized injection of drugs/reagents at optimized concentrations in each of 3 ports (mix 3 minutes, wait 2 minutes, measure 3 minutes). For the mitochondrial stress test, ports were loaded with:

1. Oligomycin (ATP synthase inhibitor) at 2μM
2. Carbonyl cyanide p-trifluoromethoxyphenyl-hydrazone or FCCP (oxidative phosphorylation uncoupler) at 0.3 μM for NB1 CMs and 0.6μM for older CMs
3. Mixture of 2μM Rotenone (respiratory complex I inhibitor) and 4μM Antimycin A (respiratory complex III inhibitor)

Real time OCR was averaged and recorded three times during each conditional cycle. OCR was determined under various conditions according to the Seahorse XF manual [17] as represented in Fig 1A. The respiratory control ratio (RCR) and coupling efficiency, two informative markers of mitochondrial function, were calculated as previously described by Brand and Nicholls [18]. Because these calculations are ratios, they are insensitive to changes in seeding densities and thus considered a good comparison of mitochondrial function with age. To calculate these ratios, the OCR for non-mitochondrial respiration was discounted. Coupling efficiency was then calculated by dividing the OCR for ATP synthesis by the OCR for basal respiration (Fig 1). The RCR was calculated by dividing the OCR for maximal respiration by the OCR for proton leak as previously described [18].

#### Glycolytic stress test

ECAR, a surrogate for anaerobic glycolysis, was measured using a Seahorse XF24 flux analyzer and primary isolated rat CMs from each maturational time point. Cells were washed with XF base medium (Seahorse Biosciences, 102353–100) and placed in a 37°C incubator without CO_2_ to degas for one hour before running the assay as shown in Fig 1B. Injection ports were loaded with optimized concentrations of the following drugs:

1. Glucose - 10mM
2. Oligomycin - 2uM to stimulate anaerobic glycolysis for ATP production
3. 2-deoxyglucose - 100mM to inhibit glycolysis

To account for the contribution of cellular respiration to acidification (ECAR) the proton pump rate (PPR) was determined as previously described by Mookerjee, et al. [19].

#### Palmitate oxidation test

Bovine Serum Albumin (BSA) conjugated palmitate was prepared as previously described [20]. OCR and ECAR were simultaneously measured using Seahorse XF analyses of primary isolated rat CMs exposed to exogenous palmitate and Etomoxir (a carnitine palmitoyl transport-1 inhibitor to stop FA supply to the mitochondria). Ports were loaded with optimized concentrations as follows:

1. Palmitate-BSA conjugate—0.15mM
2. Etomoxir—40μM
3. Etomoxir—40μM

Two doses of Etomoxir were injected to ensure maximal inhibition of exogenous FAO was obtained. PPR was calculated using methods described by Mookerjee, et al. [19] to determine the contribution of respiration and anaerobic glycolysis in response to palmitate and Etomoxir.

#### XF Analysis Validation

Experimental replicates were normalized to cell count using the same conditions for each developmental time-point. Optimization of seeding density was conducted in compliance with the Seahorse XF24 manufacturer recommendations (24) through multiple experiments to identify a cell count that provided basal OCR and ECAR readings sufficiently above background, within tested linear response ranges and with full re-oxygenation of the media attained between measurements. Validated seeding density was as follows for each maturational time point: 150,000 cells/well for NB1 CMs, 17,000 cells/well for 3 and 10 week old CMs, and 10,000 cells/well for 12–18 month old CMs. Following plating, seeding was verified by live cell imaging to demonstrate a uniform layer of CMs per well. Determining differences in metabolic responses among CMs from various time-points was done after normalization to basal ECAR and OCR. Additionally, coupling efficiency and cellular respiratory control ratio were calculated. These internally normalized measures of mitochondrial function are not dependent on seeding density so serve as a good marker of mitochondrial function comparison [18, 21]. Normalizing XF metabolic data to protein concentration would be misleading because primary isolated cardiomyocytes require an extracellular matrix protein (gelatin or laminin) for uniform plating. Normalizing to mitochondrial number does not account for differences in mitochondrial biogenesis found at different maturational time-points (highest in NB1 CMs and then declines with maturation) [22]. The selected seeding density for each maturational time-point resulted in a uniform layer of CMs per well as verified by live cell imaging prior to each assay. Optimization of drug and fuel concentrations for the selected cell seeding density and maturational age of CMs was performed to pinpoint the minimum concentrations of inhibitors that provided the maximum inhibitory effect as well as the minimum concentrations of activators that provided the maximum stimulation.

### Statistical Analysis

Statistical analyses were performed using Graph-Pad Prism 5 software (GraphPad Software, LaJolla, CA). Descriptive data are expressed as mean±SEM. Differences among different aged CMs were interrogated using a one-way ANOVA. Once statistical differences were observed, a Tukey’s post hoc test examined differences between individual groups of data where indicated. Significance was set at p<0.05.

## Results

### Mitochondrial stress test

#### Mitochondrial respiration is efficient in all age groups

**Fig 2:**
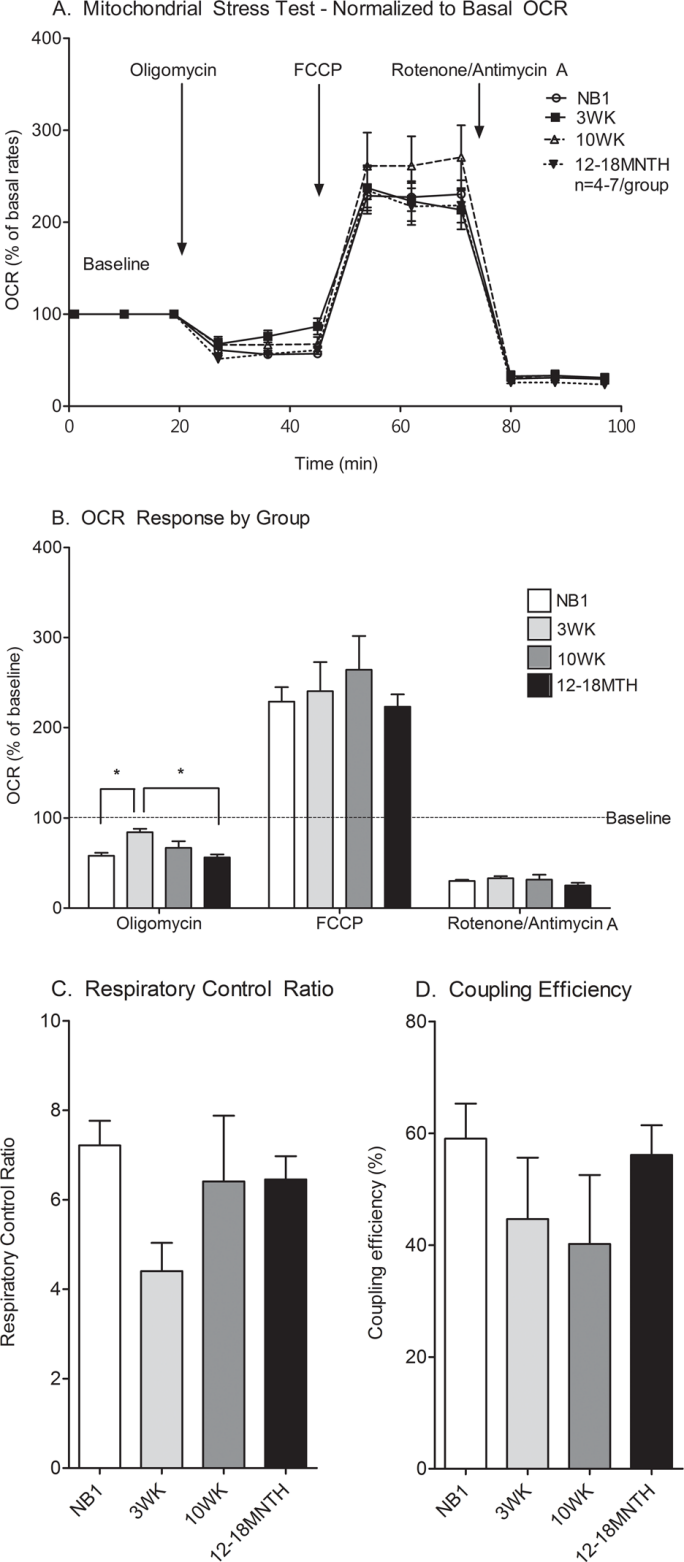
Mitochondrial stress test of cardiomyocytes at various maturational ages. XF trace (A) and bar graph (B) illustrates OCR changes (from baseline) in NB1, 3WK, 10WK, and 12–18MNTH CMs during mitochondrial stress testing. For group comparisons OCR was normalized to baseline prior to addition of Oligomycin, FCCP and Rotenone/Antimycin A where indicated. Respiratory control ratio (C) and coupling efficiency (D) as calculated from traces are compared between groups. Data are expressed as means ± SEM of 4–7 independent experiments. *p<0.05 by one-way ANOVA and Tukey’s post-test.

It was noted that basal OCR was significantly different (p = 0.017) among CMs from different maturational groups and when adjusted to cell count was lowest in the NB1 CMs (NB1 272±31pmol/min/well or *0*.*002pmol/min/cell*; 3WK 459±45pmol/min/well or *0*.*027pmol/min/cell*; 10–12WK 261±53pmol/min/well or *0*.*015pmol/min/cell*; 12–18MNTH 342±52pmol/min/well or *0*.*034pmol/min/cell*). Although this may be due to age related differences, it may also be explained by variable ATP demand or seeding density differences. Thus, metabolic response comparisons were deemed valid only after adjusting OCR to the basal respiratory rate. In doing so, we found that the % change above baseline OCR in primary isolated CMs from NB1, 3WK, 10–12WK and 12–18MNTH time points was similar during the mitochondrial stress test (Fig 2A). The OCR response to oligomycin (ATP synthase inhibitor) was higher in 3WK CMs compared to NB1 or 12–18MNTH CMs (Fig 2B). This suggests that at the 3WK time point, more oxygen is being consumed for non-ATP producing purposes (possibly driving proton leak). Neither respiratory control ratio nor coupling efficiency was significantly different between ages (Fig 2C and 2D). RCR trended lower at 3WKs (Fig 2C). The coupling efficiency, an indicator of the proportion of respiratory activity used to make ATP, also trended lower in the 3WK (44%, p = 0.19) and 10WK (40%, p = 0.13) CMs compared to NB1 (59%) and 12–18MNTH (56%) old CMs (Fig 2D).

### Glycolytic stress test

#### Glycolytic capacity is greatest in the newborn heart

**Fig 3:**
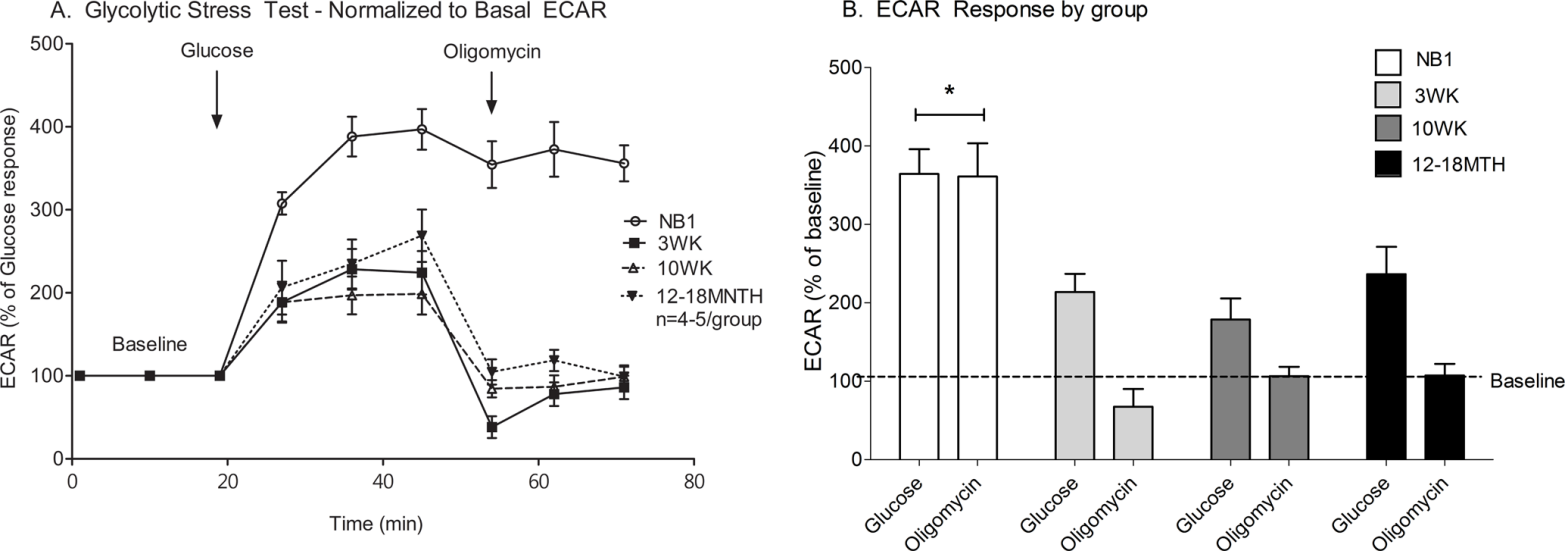
Glycolysis stress test of cardiomyocytes at various maturational ages. Extra-cellular acidification rate (ECAR) traces (A) and bar graphs (B) show the glycolytic response of NB1, 3WK, 10WK and 12–18MTH old cardiomyocytes in response to glucose and oligomycin injection where indicated. Data shown are means ± SEM of 3–5 independent experiments. *p<0.05 by one way ANOVA and Tukey’s post-test.

NB1 CMs demonstrate a more robust and sustained glycolytic capacity than more mature CMs. As illustrated in Fig 3A and 3B, ECAR response to glucose (p = 0.003) and oligomycin (p<0.0001) were different among CMs at various maturational ages. NB1 CMs have a higher glucose-stimulated ECAR and maintain the same acidification rate with inhibition of mitochondrial oxidative phosphorylation by oligomycin (364% to 361% ECAR). In contrast, 3WK (213% to 67% ECAR), 10WK (178 to 106% ECAR) and 12–18MTH (236% to 107% ECAR) CMs demonstrated a significant decrease in ECAR after oligomycin injection. This decline was most pronounced in 3WK CMs with an ECAR drop to well below baseline. The significant drop in ECAR following oligomycin injection (meant to stimulate anaerobic glycolysis) was not anticipated because oligomycin inhibits ATP synthase which typically causes cells to accelerate anaerobic glycolysis (increasing ECAR) to maintain ATP production. This led us to examine metabolic contributions of ECAR more closely.

### Proton production rate (PPR) during glycolysis

#### Lactate production from anaerobic glycolysis is lower in mature cardiomyocytes

**Fig 4:**
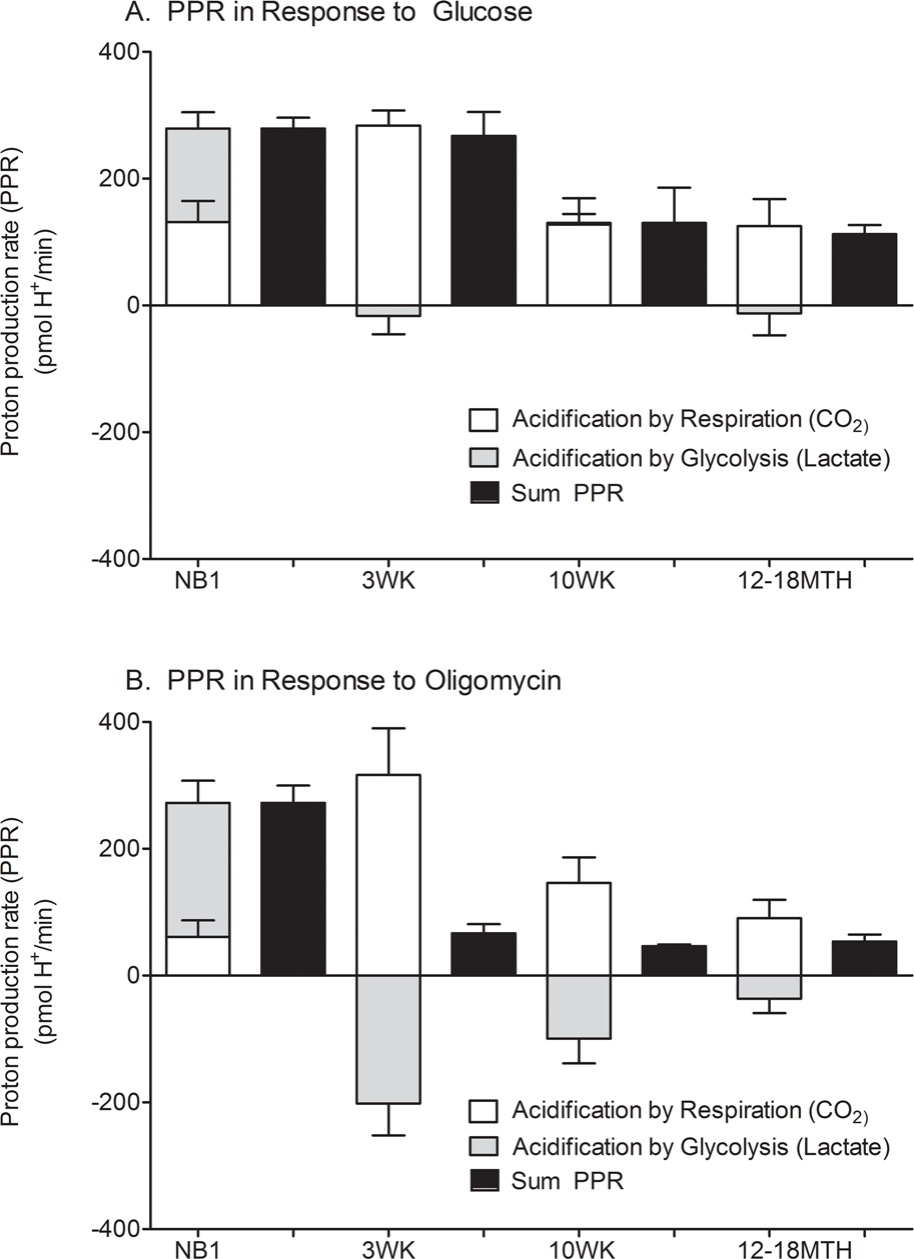
Proton production rate during glycolytic stress test. The positive or notionally negative contribution of lactate from both anaerobic glycolysis (grey) and from CO_2_/respiration (white) to the sum (black) proton production rate (PPR) are demonstrated for cardiomyocytes from each maturational time-point. The PPR is demonstrated in response to glucose (A) and oligomycin (B). Data shown are means ± SEM of 3–5 independent experiments. *p<0.05 by one way ANOVA and Tukey’s post-test.

ECAR can be affected by both lactate from anaerobic glycolysis and by CO_2_ production from the Krebs cycle. The distinct contributions to the total PPR in response to glucose (Fig 4A) and oligomycin (Fig 4B) were evaluated using equations described by Mookerjee, et al. [19]. As demonstrated in Fig 4, PPR in NB1 CMs is primarily due to lactate production from anaerobic glycolysis rather than CO_2_ production from cellular respiration. From 3 weeks of age and onward, nearly all PPR was from CO_2_ production and not lactate production. This suggests that mature CMs have a limited ability to increase ATP production from anaerobic glycolysis.

### Palmitate oxidation test

#### Fatty acid oxidation capacity is limited in neonatal cardiomyocytes

**Fig 5:**
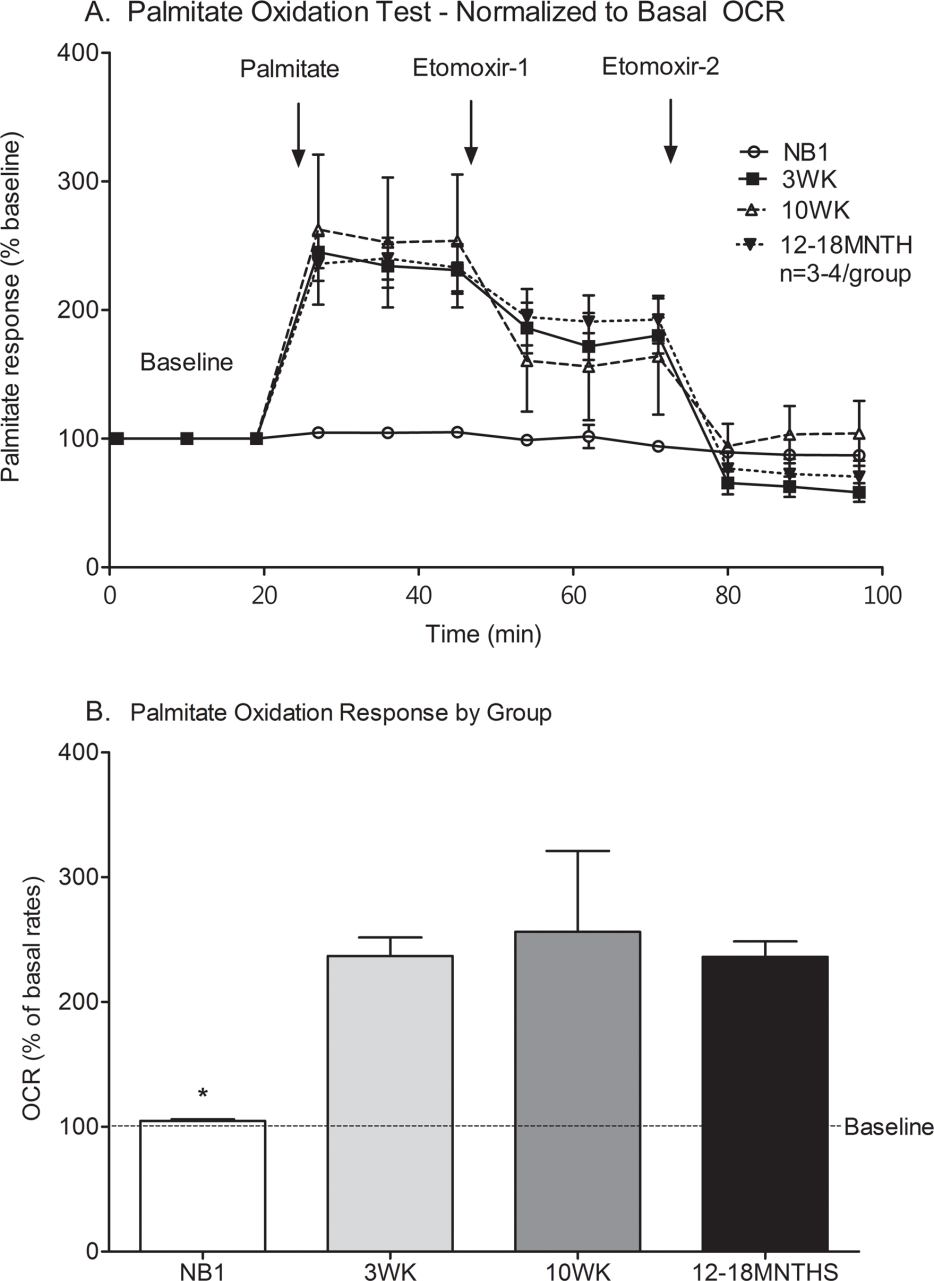
Palmitate oxidation test of cardiomyocytes at various maturational ages. OCR was measured in NB1, 3WK, 10WK, and 12–18MNTH CMs and normalized to baseline rates to interrogate differences in response among the groups. OCR response to exogenous Palmitate-BSA and Etomoxir (carnitine palmitoyl transport inhibitor) are illustrated in trace (A). Bar graphs illustrate group differences in OCR response from baseline conditions (B). Data are expressed as means ± SEM of 3–4 independent experiments. *p<0.05 by one-way ANOVA and Tukey’s post-test.

Palmitate oxidation, after normalization to baseline levels (100%), increased in response to exogenously injected palmitate to 105%, 237%, 256% and 236% in NB1, 3WK, 10WK and 12–18MTH respectively (Fig 5). NB1 CMs have a decreased ability to oxidize exogenous palmitate compared to 3WK, 10 WK or 10–12MTH old CMs (p≤0.01). There was no difference in palmitate oxidation among CMs from the older time points (3WK, 10–12WK or 12–18MTH) on post-hoc analyses.

### Proton production rate (PPR) during palmitate oxidation

#### CO_2_ production is the primary contributor to ECAR in mature cardiomyocytes

**Fig 6:**
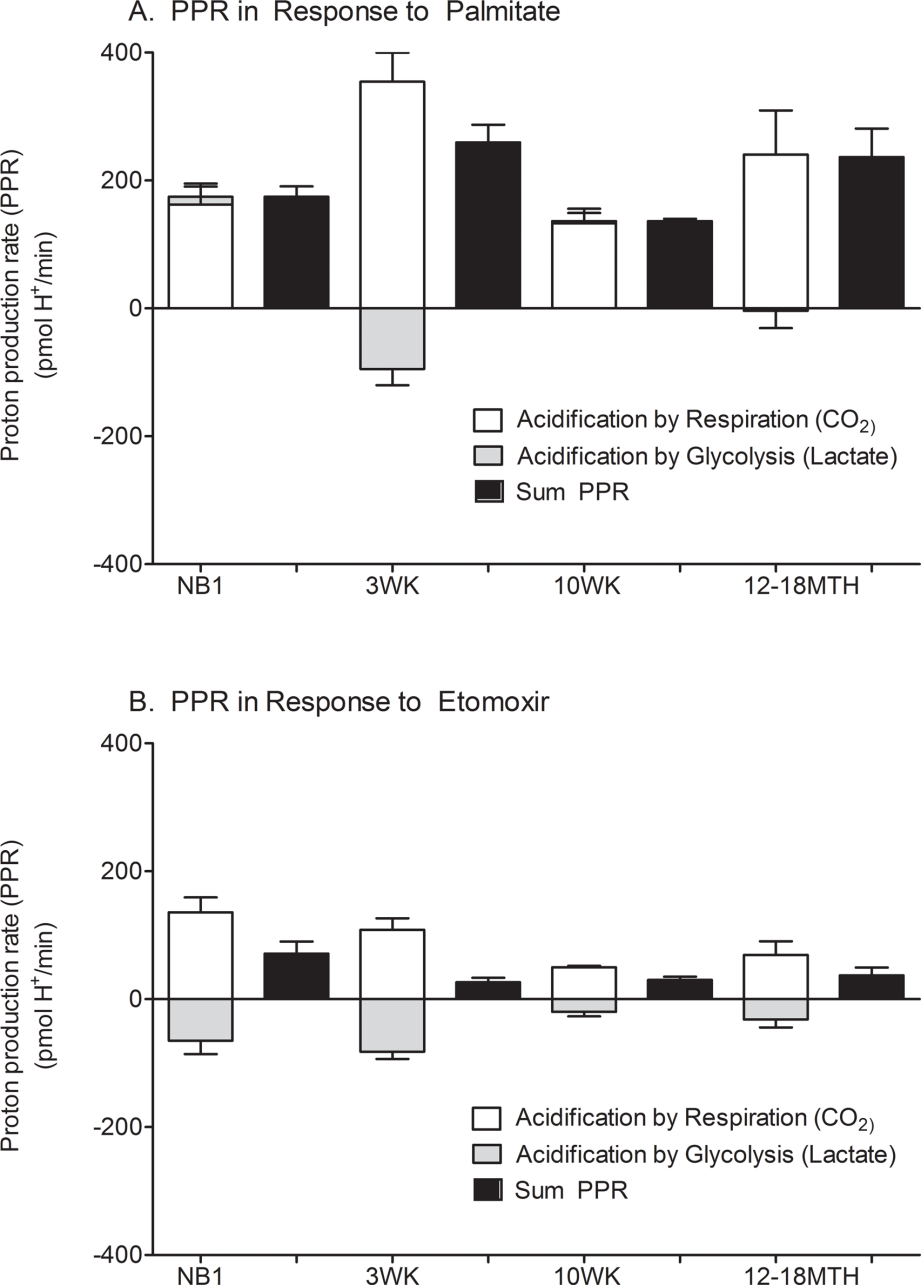
Proton production rate during palmitate oxidation test. The positive or notionally negative contribution of lactate from both anaerobic glycolysis (grey) and from CO_2_/respiration (white) to the sum (black) proton production rate (PPR) are demonstrated for cardiomyocytes from each maturational time-point. The PPR is demonstrated in response to palmitate (A) and etomoxir (B). Data shown are means ± SEM of 3–5 independent experiments. *p<0.05 by one way ANOVA and Tukey’s post-test.

Both ECAR and OCR were measured simultaneously during the palmitate stress test so the contribution of CO_2_ from the Krebs cycle to the PPR during FAO could be calculated [19]. As demonstrated in Fig 6A, the PPR in response to palmitate is solely from the Krebs cycle/cellular respiration. Interestingly, although NB1 CMs have a relative inability to oxidize palmitate, they had an increase in non-glycolytic PPR in the presence of Etomoxir. Perhaps this was due to oxidation of other endogenous fuels.

## Discussion

Ageing and CVD are highly interconnected and increasing evidence suggests a metabolic pathogenesis [23]. In order to develop studies focused on prevention and therapeutics for metabolically-regulated cardiac health and disease, it is imperative to understand normal CM metabolism at various ages using similar experimental conditions and methods used to investigate disease. This study establishes investigative methods and normal cellular bioenergetics profiles of healthy rat CMs at various developmental time points using XF analyses.

Neonatal CMs are very different from mature CMs in morphology (kite vs. rod shaped), nucleation (single vs. bi-nucleated until postnatal day 5–8), ploidity (polyploidal until 7 days) and the ability to proliferate (markedly decreased by 3WK) [24]. Cardiac mitochondria are very dynamic with continuous turnover and normal fluctuations at various stages of maturation. Indeed, cardiac mitochondrial biogenesis markedly increases in the perinatal time period followed by maturation and redistribution into tightly packed groups between developing sarcomeres by 3WK of age [22]. So it only makes sense that fuel metabolism is also different at these time points.

The fetal heart with its lower cardiac demand, limited oxygen supply and continuous fuel source from maternal circulation is tailored for anaerobic metabolism [25]. In contrast, the normal resting adult heart preferentially metabolizes fatty acids which consumes more oxygen but yields more ATP per molecule [8, 25]. Using instrumented fetal lambs to measure myocardial fuel consumption, Fisher et al. found that while *in utero* the developing heart consumes more glucose, lactate and pyruvate than the adult heart [26]. Measuring metabolic enzyme activity in fetal lamb hearts, Ohtsuka et al. found that lactate dehydrogenase and pyruvate kinase (markers of glycolysis) were 3 to 4 fold higher than citrate synthase activity (marker of oxidative phosphorylation) and that citrate synthase activity was significantly higher in adult sheep hearts compared to the fetal lamb [27]. Isolated fetal pig hearts have a relative inability to oxidize palmitate [28]. Using perfused isolated rabbit hearts, Lopashuk et al. found that in newborns 44% of steady-state ATP production was from glycolysis, 25% from lactate, 18% from exogenous glucose and only 13% was from palmitate, but by 7 days of age glycolysis decreased and palmitate oxidation increased significantly [29]. Our study reproduced these findings using the increasingly popular method of real-time XF analyses and confirmed these age-related metabolic differences at a cellular level in isolated cardiomyocytes.

XF analysis is a powerful tool because it allows real-time measurement of ECAR and OCR using intact cells under stimulating and inhibiting conditions. Analysis by this method can provide metabolic data on a wide variety of cells including primarily isolated cells from small animal models (even NB1 rodents) without radioisotope labeling or laborious perfusion methods. Other methods to measure cellular respiration include using Clark electrodes and isolated mitochondria. Although this is an extremely useful tool, the method is time consuming and has some additional limitations:

1. Isolated mitochondria lack other cellular structures including cytoplasmic fuels stores/lipid droplets and membrane transport and signaling proteins that may play a role in cellular metabolism
2. Metabolic interactions between glycolysis and oxidative phosphorylation cannot be tested at the same time
3. Changes in cellular respiration due to altered mitochondrial content (biogenesis or mitophagy) could be lost

Recently, XF analysis has emerged as a highly utilized method to assess cellular glycolytic and respiratory capacity in real time. Methods have been previously tested in isolated rodent CMs [20] and use is increasingly popular. Our study defines normal cellular bioenergetics profiles at various stages of maturation and validates methods to allow investigation of age-appropriate metabolic differences using XF analyses of isolated CMs. This study was necessary to advance studies of developmental programming of cardiac disease and to highlight the need for careful study design and data interpretation so that appropriately aged CMs are used to investigate metabolic pathogenesis of disease. For example, if cells have a preference for anaerobic metabolism to make ATP, they may be resistant to oxidative injury. This is highlighted by Sansbury, et al. who found that adult CMs respond more quickly to oxidative stress than neonatal CMs [30]. Therefore, by appropriately identifying which component of cellular bioenergetics is responsible for the pathology, the experimenter can develop specific therapeutic strategies aimed at treating CVD [5, 31, 32].

Our findings confirm that neonatal rat CMs have a very different metabolic profile than adult CMs by real time XF analyses. Normal neonatal CMs demonstrate a glycolytic preference as shown by a robust and sustained glycolytic capacity, an ECAR almost exclusively from lactate production and a diminished ability to oxidize exogenous palmitate. On the other hand, adult CMs demonstrate a greater preference for oxidative phosphorylation. At the 3WK time point (weaning), primary isolated CMs respond to both glucose and palmitate; however using CMs from this time point should be done with caution because they demonstrate a higher OCR for non-ATP production and have a trend to lower respiratory control ratio (a marker of mitochondrial function).

Additional findings in this study highlight the need for caution in interpreting the standard glycolytic stress test. Recent advances in XF technology have demonstrated that the contribution of glycolysis and respiration to total PPR varies considerably between different cell types [19]. In fact, although ECAR may increase with glucose provision this may be due to cellular respiration, rather than lactate production from anaerobic glycolysis [19]. Conducting a post-analysis assessment of both lactate and CO_2_ contribution to the PPR (total ECAR) provided a great deal of insight into the metabolic profiles of CMs. In doing so, we found that respiration was the sole contributor to PPR in adult CMs and that ECAR actually decreased (rather than increased as expected) with oligomycin “stimulation” of glycolysis. These findings highlight the importance of understanding specific cell type and age related bioenergetic profiles and that making assumptions about glycolytic capacity without accounting for the contribution of respiratory CO_2_ is fraught with misinterpretation.

### Limitations

It is important to recognize that isolation of CMs from their *in vivo* environment may affect their bioenergetics. Choices of culture media (including differences in fuels like glucose, pyruvate, amino acids) as well as incubation conditions (oxygen, CO_2_ and temperature) also need to be carefully considered and controlled. Another limitation to using XF analyses as a sole marker for cardiac metabolism is that it represents cellular metabolism but cannot readily account for blood flow, fuel supply, metabolic needs of additional cells in the heart or cardiac workload. Despite these significant differences, most studies are interested in CM metabolism. It should be recognized that the whole heart also contains non-myocytes (fibroblasts and endothelial cells). When assessing CM bioenergetics, precautions should be taken to ensure a relative absence of these non-myocytes. Although we took additional steps to separate CMs from other cardiac cells, the possibility of culture contamination should be recognized. Additional limitations to our study include the inability to interpret basal metabolic rate due to differences in seeding density between NB1 and older CMs and the inability to normalize to protein concentration due to the need for plating onto gelatin or laminin coated wells. CMs did not lay down in a uniform manner without an extracellular matrix and when the cells coalesced the ECAR and OCR measurements were not accurate. Over the course of three years we have replicated XF analyses values within a fairly narrow range when using the described methods, seeding density and drug concentrations for the outlined time points.

## Conclusion

In conclusion, our study used XF analysis to directly compare differences in glycolytic capacity, mitochondrial respiration and palmitate oxidation in primary isolated rat CMs at four developmental time points. In doing so, we confirmed that significant developmental differences in fuel metabolism at birth and adulthood. Based upon these findings, we believe that caution should be taken to assure that appropriate maturational time points are used to investigate mitochondrial dysfunction as a cause of cardiac disease. Specifically, XF analyses of newborn CMs should be reserved to study fetal/neonatal or developmentally programmed disease and older CMs (≥10 weeks) should be used to investigate adult cardiac disease pathogenesis. Accounting for the contribution of cellular respiration and CO_2_ production to the total ECAR is necessary in interpreting glycolytic capacity, especially in adult CMs. These findings highlight the need for a heightened awareness of maturational time points when drawing conclusions about cardiac disease related to mitochondrial dysfunction.
